# The role of impaired adipogenesis in insulin resistance among non-obese individuals

**DOI:** 10.3389/fphys.2025.1739215

**Published:** 2026-01-07

**Authors:** Shamma Almuraikhy, Maha Alser, Khaled Naja, Najeha Anwardeen, Samir Taha, Jomon John, Sharique Halim, Saif Badran, Mohamed Badie Ahmed, Salma Jarrar, Ghanem Aljassem, Fatima Saoud Al-Mohannadi, Suhail Doi, Asmaa Abdel-Aziz, Yousra Jalal Elaf, Mohamed A. Elrayess

**Affiliations:** 1 Biomedical Research Center, Qatar University, Doha, Qatar; 2 Oral and Maxillofacial Surgery, Al-Wakra Hospital, Hamad Medical Corporation, Doha, Qatar; 3 Mahatma Gandhi Cancer Hospital (MGCH) Miraj, India; 4 Division of Plastic and Reconstructive Surgery, Washington University School of Medicine, Saint Louis, MO, United States; 5 Plastic Surgery Department, Hamad General Hospital, Hamad Medical Corporation, Doha, Qatar; 6 College of Medicine, QU Health Cluster, Qatar University, Doha, Qatar; 7 Department of Biomedical Science, College of Health Sciences, Qatar University, Doha, Qatar

**Keywords:** adipogenesis, insulin resistance, metformin, non-obese, tumor necrosis factor alpha

## Abstract

**Introduction:**

Adipogenesis is an essential process for energy storage, hormone regulation, and overall metabolic health. Previous work showed that impaired adipogenesis plays an important role in the development of obesity-associated insulin resistance. This project investigates the role of impaired adipogenesis in the development of insulin resistance among non-obese (lean/overweight) individuals under physiological and pathological microenvironments.

**Methods:**

Subcutaneous adipose tissue samples were obtained from insulin-sensitive and insulin-resistant non-obese cohorts undergoing maxillofacial or body contouring surgeries. Preadipocytes were isolated and examined for proliferation and adipogenic capacity, insulin signaling, and inflammatory markers. These assessments were conducted under basal conditions and following treatment with either tumor necrosis factor alpha (TNF-α) to induce insulin resistance or metformin to promote insulin sensitivity.

**Results:**

Insulin-resistant participants, in comparison to insulin-sensitive counterparts, showed lower adipogenic capacity, higher susceptibility to the anti-adipogenic and pro-inflammatory effect of TNF-α potentially due to hyperphosphorylation of insulin receptor substrate-1.

**Discussion:**

This highlights the role of impaired adipogenesis in the pathogenesis of insulin resistance among non-obese individuals. Further research is needed to understand the impact of impaired adipogenesis and the potential therapeutic interventions targeting adipogenesis to improve insulin sensitivity in non-obese individuals.

## Introduction

Adipose tissue is a specialized type of connective tissue primarily made up of adipocytes, which function to store excess energy in the form of lipids when energy intake surpasses energy expenditure (Zwick et al., 2018). Additionally, adipose tissue acts as a dynamic endocrine organ, releasing and reacting to various peptides and steroids, thereby playing a crucial role in metabolic regulation and overall physiological balance (Choe et al., 2016). Adipogenesis is the process by which preadipocytes undergo differentiation and maturation into fully functional adipocytes (Rosen and MacDougald, 2006). It starts with the differentiation of precursor cells that resemble fibroblasts into lipid-laden endocrine cells, that are capable of responding to insulin by initiating glucose uptake and lipogenesis, as well as leptin secretion, which helps in energy intake regulation (Sarjeant and Stephens, 2012).

The adipogenesis process is complex and tightly regulated by various transcription factors, hormones, and signaling molecules. At the molecular level, adipogenesis is facilitated by several transcription factor families, including the master regulators: the peroxisome proliferator-activated receptor gamma (PPARγ) and the CCAAT enhancer-binding protein alpha (C/EBPα). These transcription factors control the differentiation of preadipocytes into mature adipocytes and enhance their lipid accumulation (Madsen et al., 2014). Subcutaneous adipose tissue (SAT) plays an essential role in our metabolism by its capacity to store excess energy in the form of lipids, preventing their accumulation in metabolically unfavorable ectopic sites (Gustafson et al., 2015). When SAT adipogenesis is impaired, this manifests as limited expansion capacity (hyperplasia) and an increase in adipocyte size (hypertrophy). This in turn results in an increase in fat deposition at visceral and ectopic sites, recruitment of inflammatory cells, and expression of an insulin-resistant phenotype characterized by a reduced glucose transporter type 4 (GLUT4) content (Longo et al., 2019). Furthermore, adipocyte hypertrophy is characterized by elevated expression and secretion levels of adipokines, which contribute to the development and progression of metabolic disorders (Clemente-Suárez et al., 2023).

Previous studies showed clear evidence of impaired adipogenesis in the pathogenesis of obesity-induced insulin resistance (Almuraikhy et al., 2016; Smith and Kahn, 2016; McLaughlin et al., 2007). However, the role of SAT-impaired adipogenesis in insulin resistance among non-obese individuals remains unclear. Another area of interest is the differential impact of SAT sites on metabolic health. These studies have been challenged by the logistic difficulty of acquiring SAT samples, especially among non-obese individuals. In this study, SAT fat samples were collected either from the thigh fat stores or buccal fat pads (BFP). The latter presents a richly vascularized tissue housing a stem cell population that exhibits a comparable differentiation capacity and phenotype to fat stem cells found in abdominal SAT (Farré-Guasch et al., 2010).

This study investigates the role of impaired adipogenesis in the pathogenesis of insulin resistance among non-obese individuals and examines the impact of insulin sensitivity/resistance modulators on this phenotype by administrating tumor necrosis factor-alpha (TNF-α), known to promote insulin resistance, and metformin, a drug known to improve insulin sensitivity.

## Materials and methods

### Cohort

Fourteen lean and overweight individuals (BMI ≤30 kg/m^2^) undergoing maxillofacial or body contouring surgery at Hamad Medical Corporation (HMC) were recruited. Fat samples were analyzed at Qatar University (QU). Approvals of the Institutional Research Board (IRB) committees for both institutions (HMC and QU) were sought before the onset of research (MRC-03- 21-154/MRC-01-20-466 and QU-IRB 1548-EA/21/QU-IRB 1412-EA/2, respectively). Participants were excluded if they were diabetics on medications or had been diagnosed with cardiovascular disease, prior bariatric surgery, cancer, glaucoma, macular degeneration, or any form of coagulopathy. Written informed consent was obtained from all subjects before surgery. Baseline blood investigations including fasting plasma glucose (FPG), lipids profile, and liver function enzymes were measured at HMC using a standard chemistry analyzer (Cobas; Roche Diagnostics, Mannheim, Germany). Insulin resistance was measured using the Homeostatic Model Assessment for Insulin Resistance (HOMA-IR) (Grundy et al., 2004; Minh et al., 2021) calculated according to the formula below, with a cut-off of 1.85 being used to define insulin resistance (Lin et al., 2022; Esteghamati et al., 2009; Gayoso-Diz et al., 2013; Almuraikhy et al., 2023).

$$\text{HOMA}-\text{IR}=\frac{\text{Fasting}\;\text{glucose}\;\left(\text{mmol}/L\right)\times \text{fasting}\;\text{insulin}\;\left(\text{mU}/L\right)}{22.5}$$

Subcutaneous adipose tissue biopsies (0.5–1 gram) were obtained from either the BFP during maxillofacial surgery or from thighs during body contouring procedures.

### Isolation of stromal vascular fraction cells from human SC adipose tissue

Stromal vascular fractions (SVFs) were isolated from BFP tissue and thigh biopsies collected from the recruited subjects as previously described (Elrayess et al., 2017). SVFs were resuspended in stromal media containing DMEM-F12 with 10% FBS, 1% Antibiotic-Antimycotic solution, and 1% L-Glutamine (200 mM). The cells were then maintained in a humidified incubator at 37 °C with 5% CO_2_. The media were changed every 2–3 days until the cells reached 80%–90% confluence. Once confluent, the cells were frozen.

### Proliferation, differentiation and adipogenic capacity

To measure cell proliferation, SVF-derived preadipocytes from each depot were separately cultured in the stromal medium for 4 days. Cells were then fixed and stained with 4′,6-diamidino-2-phenylindole (DAPI) (Molecular Probes by Life Technologies, D1306) to quantify nuclei in 25 fields per well using an automated imaging system (Cytation 5, Agilent Technologies, Santa Clara, CA, United States). The proliferation rate was determined by calculating the percentage of total cells compared to untreated controls. Differentiation was induced by culturing preadipocytes in a stromal medium overnight, followed by 7 days in a differentiation medium containing (in DMEM/Ham’s F-12): 3% fetal bovine serum, 0.25 mM methylisobutylxanthine (IBMX), 66 µM biotin, 5 µM rosiglitazone, 1 µM dexamethasone, and 200 nM human insulin. On day 7, the medium was switched to a maintenance medium (identical to the differentiation medium but lacking IBMX and rosiglitazone) for an additional 10–14 days. Cells were then fixed with 4% formaldehyde (Thermo Scientific, Waltham, MA, United States, 28,908) and stained with DAPI and Lipidtox (Invitrogen, H34476) to identify nuclei and differentiated adipocytes, respectively. The Cytation 5 Cell Imaging Multi-Mode Reader (Agilent Technologies) automatically quantified the total number of nuclei (DAPI-positive) and differentiated adipocytes (Lipidtox-positive) in 25 fields per well. Adipogenic capacity was assessed by calculating the percentage of Lipidtox-positive cells relative to the total number of nuclei.

### Preadipocyte treatment with TNF-α and metformin

SVF-derived preadipocytes from each depot were exposed to either TNF-α or metformin throughout the proliferation and differentiation/maintenance periods. Recombinant human TNF-α protein (210-TA-100, R&D Systems) was added at a final concentration of 10 ng/mL. Metformin was used at a final concentration of 1 mM in combination with insulin. The culture medium was changed every 2–3 days to maintain optimal conditions and treatment exposure.

### Measurement of secreted cytokines

Media supernatants of preadipocyte cultures were collected following the completion of differentiation. Accumulated levels of secreted cytokines (IL-1 beta, IL-1RA, IL-6, IL-8 (CXCL8), IL-10, IL-22, MCP-1 (CCL2), TNF alpha) in the last 4 days before cell fixation were measured using Inflammatory Cytokine Human ProcartaPlex Mix&Match 8-plex (catalogue No: PPX-08-MXRWF7P) according to manufacturer’s instructions and assessed by Luminex Flexmap 3D using xPONENT 4.2 software. Cytokine levels in the secreted medium (from 96-well plate) were normalized to the total number of cells in each well, as determined by DAPI staining. This will allow us to express the cytokine levels relative to the cell number in each well. The normalization formula is shown below:

$$\text{Normalized Cytokine Level}=\frac{Cytokine\;Concentration\;in\;Medium\;}{Total\;Cell\;Count\;per\;Well\;}$$

### Assessment of insulin signaling pathway

The assessment of the insulin signaling pathway was conducted by measuring the serine phosphorylation levels of IRS-1, 70S6K, GSK3α/β, BAD, PTEN, AKT, S6RP, and mTOR in total cell lysates, using a commercial Bio-Plex Pro™ Cell Signaling Akt Panel (Bio-Rad, Heracles, CA, United States) and assessed by Luminex 200 technology (Thermo Fisher Scientific, Waltham, MA, United States), according to the manufacturer’s instructions. This is a magnetic bead-based immunoassay that employs a multiplex format, allowing for the simultaneous detection and quantification of multiple proteins involved in critical intracellular signaling pathways. This approach enhances measurement robustness and sensitivity compared to traditional methods.

### Statistical analysis

All statistical analyses were performed using GraphPad Prism (v. 10.1.0). Normality of variables was assessed using the Shapiro-Wilk test. Group comparisons were made using repeated measures of one-way ANOVA for untreated, metformin, and TNF-alpha-treated groups. Mann-Whitney U tests or Student’s t-tests were used to compare between insulin-sensitive and insulin-resistant groups. A power analysis was conducted to determine the required sample size for detecting differences in adipogenesis between IR and IS individuals. Based on previous studies (Almuraikhy et al., 2016), we anticipated a 30% greater impairment in adipogenesis in IR subjects compared to matched IS controls. Using G*Power 3.1 software (Faul et al., 2007), we calculated the sample size needed to detect a large effect size (Cohen’s d = 0.8) with 80% power at a significance level of α = 0.05 for a two-tailed independent samples t-test. The analysis indicated that a total sample size of 14 participants (7 per group) would be sufficient to detect significant differences between groups. The required variance, based on an estimated standard deviation of 0.375, was calculated to be approximately 0.1406.

## Results

### General characteristics of study population

Fourteen healthy non-obese (BMI 20–30 kg/m^2^), aged 18–45 years old individuals undergoing maxillofacial surgery or body contouring procedure at HMC were recruited. Participants were dichotomized into IS and IR groups based on their HOMA-IR using 1.85 as cut-off. Accordingly, 57% were insulin sensitive (IS) and 43% were insulin resistant (IR) (Table 1). Baseline characteristics revealed no significant differences in gender distribution or kidney function markers (urea, creatinine). However, the IR group had a significantly higher BMI compared to the IS group. This difference was accompanied by higher fasting blood sugar and HOMA-IR, a marker of insulin resistance. Furthermore, the IR group displayed an altered lipid profile, with higher triglycerides and lower high-density lipoprotein (HDL) cholesterol compared to their IS counterparts.

**TABLE 1:** General characteristics of study population.

Variable	Insulin-sensitive (n = 8)	Insulin-resistant (n = 6)	P value
Gender (F: female, M: male)	F: 5, M: 3	F: 5, M: 1	0.58
Depot (T: Thigh, B=Buccal fat pad)	T: 4 B:4	T: 4 B: 2	0.53
Age	24.5 (24–31)	37 (29–44)	0.055
Weight (kg)	62.11 (14.49)	75.13 (14.2)	0.12
Height (cm)	77.46 (1.62–156.88)	158.35 (152.42–166.6)	0.22
BMI (kg/m^2^)	23.61 (3.7)	27.56 (1.88)	0.025
Fasting blood sugar (mmol/L)	4.65 (0.29)	5.14 (0.59)	0.10
Insulin (mU/L)	5.36 (1.89)	16.37 (7.76)	0.016
HOMA-IR	1.21 (0.47)	3.14 (0.93)	0.058
Urea (mmol/L)	3.9 (0.74)	3.88 (1.25)	0.97
Creatinine (μmol/L)	59.88 (13.53)	61.67 (17.31)	0.83
Sodium (mmol/L)	140 (137.5–140)	139 (138.25–139)	0.60
Potassium (mmol/L)	4.13 (0.4)	4.47 (0.31)	0.11
Chloride (mmol/L)	104.2 (1.1)	102.73 (2.16)	0.17
Bicarbonate (mmol/L)	24 (24–25)	24 (22–26)	1.000
Calcium (mmol/L)	2.29 (0.09)	2.32 (0.15)	0.64
Total protein (g/L)	69.25 (2.87)	70.2 (4.44)	0.71
Albumin (g/L)	38.83 (3.6)	38.33 (5.28)	0.85
Uric acid (µmol/L)	278.25 (56.54)	268 (49.67)	0.78
ALT (U/L)	14 (4.31)	16 (6.9)	0.55
AST (U/L)	21.43 (6.05)	15.17 (2.32)	0.035
Cholesterol (mmol/L)	4.08 (0.6)	4.27 (0.56)	0.55
Triglycerides (mmol/L)	0.69 (0.2)	1.1 (0.28)	0.015
HDL (mmol/L)	1.75 (0.6)	1.22 (0.43)	0.19
LDL (mmol/L)	2.16 (0.48)	2.57 (0.52)	0.16

Data are expressed as mean (standard deviation) or median (interquartile range), depending on the distribution assessed using the Shapiro–Wilk normality test.

### Comparing proliferation and adipogenic capacity between thigh and BFP derived preadipocytes

**FIGURE 1:**
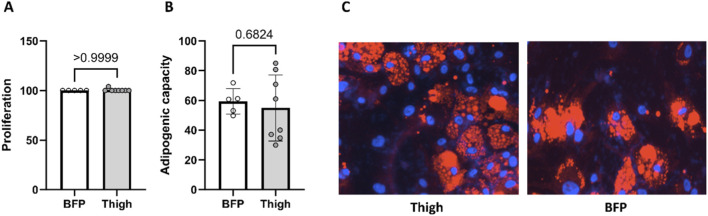
Comparison of the proliferation percentage **(A)** and adipogenic capacity **(B)** between thigh and buccal fat tissue. **(C)** Immunofluorescence imaging: Nuclei stained with DAPI (blue) and lipid droplets visualized using LipidTOX (red).

The efficiency of proliferation and differentiation of expanded preadipocyte cultures were compared between thigh and BFP derived subcutaneous preadipocytes. The proliferation percentage (Figure 1A) and the adipogenic capacity (Figure 1B) of thigh-derived preadipocytes showed no significant difference compared to preadipocytes derived from buccal fat tissue. Immunofluorescence imaging are also shown (Figure 1C). Data from both depots will be pooled in further analyses to enhance statistical power for identifying differences between insulin-sensitive and insulin-resistant states as well as treatment effects.

### Comparing preadipocyte proliferation in IS vs. IR individuals and the effects of TNF-α/Metformin treatment

**FIGURE 2:**
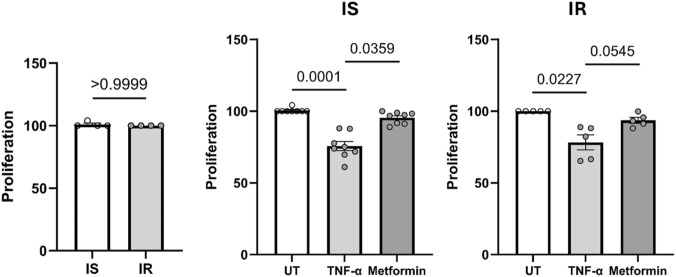
Proliferation of preadipocytes, pooled from both depots, from insulin sensitive (IS) and insulin resistant (IR) individuals, and effect of TNF-α/metformin + insulin on preadipocyte proliferation percent in both groups. Data are presented as mean ± SEM; Differences between IS and IR were tested by independent-sample t-test; *p < 0.05. The proliferation percentage was normalized to untreated cells.

Preadipocytes derived from both IS and IR individuals exhibited no significant difference in proliferation under normal conditions (Figure 2). Treatment of preadipocytes with TNF-α for 4 days (Figure 2) resulted in significant inhibition of proliferation compared to untreated controls in both IS and IR-derived preadipocytes (p < 0.05). Treatment with metformin + insulin showed a slightly reduced proliferation compared to untreated control in IS and IR derived preadipocytes.

### Comparing preadipocyte differentiation in IS vs. IR individuals and the effects of TNF-α/Metformin treatment

**FIGURE 3:**
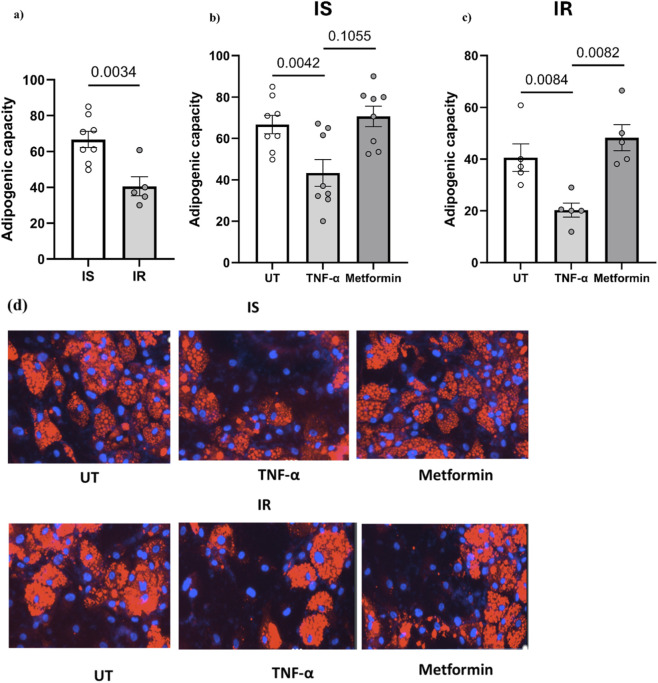
**(a)** Adipogenic capacity of differentiated preadipocytes pooled from both depots derived from IS and IR lean/overweight individuals. **(b,c)** Effect of TNF-α/metformin + insulin on preadipocyte differentiation. **(d)** Immunofluorescence imaging: Nuclei stained with DAPI (blue) and lipid droplets visualized using LipidTOX (red). Data are presented as mean ± SEM; Differences between IS and IR were tested by independent-sample t-test; *p < 0.05.

The preadipocytes derived from all IR individuals exhibited reduced adipogenic capacity compared with their IS counterparts (by 38%, p = 0.0034) (Figure 3a). Among different cytokines, we selected TNF-α to confirm the effect of the proinflammatory cytokines on the differentiation of preadipocytes derived from individuals with insulin sensitivity and insulin resistance. In TNF-α treated cells, results showed reduced adipogenic capacity in preadipocytes derived from both IS and IR individuals (p < 0.05) compared to untreated cells (Figures 3b,c). Treatment of preadipocytes with metformin + insulin showed only significant improvement of adipogenic capacity in IR individuals (p < 0.05), but not in their IS counterparts, compared to the capacity of untreated cells (Figures 3B,C). Immunofluorescence imaging are also shown (Figure 3d).

### Comparing cytokine secretion and insulin signaling in preadipocytes from IS vs. IR individuals and the effects of TNF-α/Metformin treatment

**FIGURE 4:**
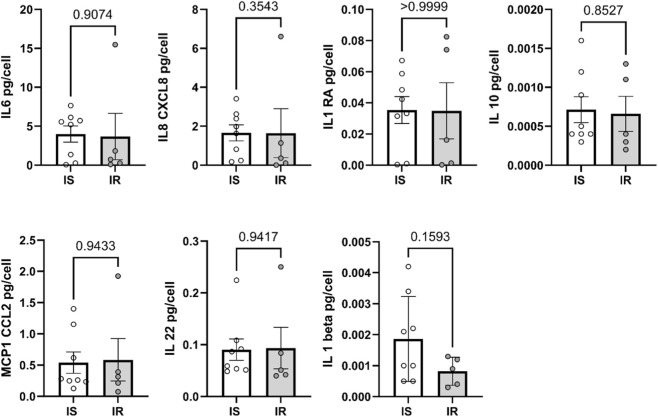
Secreted levels of IL-6, IL-8, IL-1RA, IL-1 beta, IL-10, IL-22, MCP-1 were compared between IS and IR-derived differentiated adipocytes pooled from both depots. Cytokine levels were normalized by dividing cytokine levels to the cell number. Data are presented as mean ± SEM; Differences between IS and IR were tested by independent-sample t-test; *p < 0.05.

**FIGURE 5:**
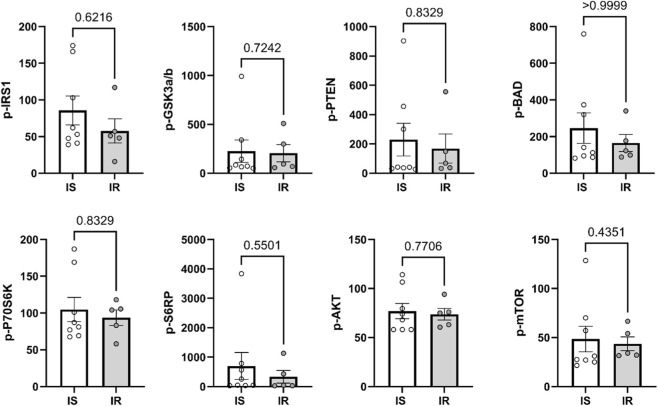
Level of phosphorylated IRS-1, 70S6K, GSK3α/β, BAD, PTEN, AKT, S6RP, and mTOR were measured in differentiating adipocytes derived from insulin sensitive (IS) and insulin resistant (IR) individuals pooled from both depots. Data are presented as Mean ± SEM. *P < 0.05, Independent and paired samples t-test.

To explore the potential molecular mechanisms underlying proliferation and differentiation in preadipocytes from IS and IR individuals, levels of secreted cytokines in differentiated adipocytes were compared. These cytokines included IL-6, IL-8 (CXCL8), IL-1RA, IL-1β, IL-10, IL-22, and MCP-1 (CCL2). No significant differences in cytokine secretion were observed between differentiated adipocytes derived from IS and IR individuals (Figure 4), although, a trend towards lower IL-1β secretion was found in IR-derived adipocytes. Insulin signaling pathways were further assessed by analyzing the phosphorylation levels of key proteins involved in the pathway, including IRS-1, 70S6K, GSK3α/β, BAD, PTEN, AKT, S6RP, and mTOR, in differentiated adipocytes from both groups (Figure 5). No significant differences were detected in the phosphorylation levels of these proteins between differentiated adipocytes from IS and IR individuals. Summary data for insulin signaling are presented in Supplementary Table S1.

### The effect of TNF-α or metformin on the level of secreted cytokines in IS and IR differentiated preadipocytes

**FIGURE 6:**
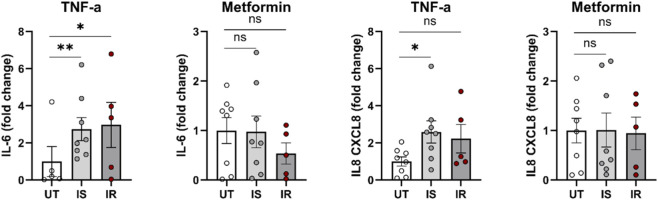
Level of secreted IL-6 and IL-8 in IS and IR differentiated preadipocytes pooled from both depots and derived from lean/overweight individuals and after treatment with TNF-α or metformin. Cytokine levels were normalized by dividing cytokine levels to the cell number and then normalized to the mean of untreated group. Data are presented as mean fold change ±SEM; Differences were tested by ANOVA/Kruskal Wallis followed by *post hoc*Friedman/Dunnett test; *p < 0.05, **p < 0.01.

Differences in secreted cytokine levels were compared between IS and IR differentiated adipocytes in response to chronic treatment with TNF-α or metformin + insulin. Results indicated that TNF-α treatment significantly increased IL-8 secretion in IS-derived adipocytes (p < 0.05), but not in IR-derived cells (Figure 6). Additionally, TNF-α treatment resulted in higher IL-6 secretion in both IR and IS adipocytes, with a more pronounced effect observed in IS cells. Other cytokines did not show significant differences (Supplementary Figure S1).

### The effect of TNF-α and metformin treatment on insulin signaling pathway in IS and IR differentiated preadipocytes

**FIGURE 7:**
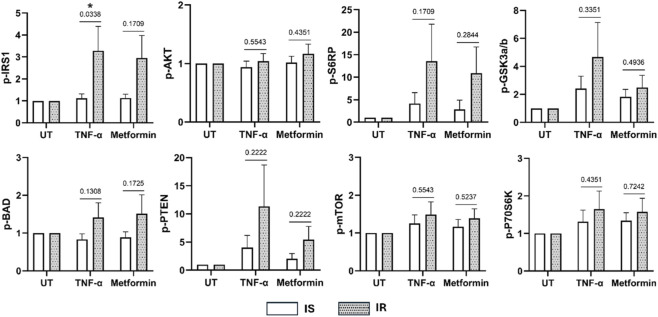
Insulin signaling pathway of differentiated adipocytes pooled from both depots and derived from IS and IR lean/overweight individuals after treatment with TNF-α or metformin + insulin. Fold changes in response to TNF-α or metformin treatment are shown. Data are presented as mean ± SEM; Differences between IS and IR were tested by independent-sample t-test; *p < 0.05.

Differences in the insulin signaling pathway (IRS-1, 70S6K, GSK3α/β, BAD, PTEN, AKT, S6RP, and mTOR) were compared between IS and IR differentiated adipocytes in response to long-term treatment with TNF-α or 1 mM metformin + insulin (Figure 7). IR-derived differentiated preadipocytes exhibited significantly higher levels of phosphorylated IRS-1 (p < 0.05) compared to IS after treatment with TNF-α. However, TNF-α and metformin treatment did not cause significant changes in the activation levels of 70S6K, GSK3α/β, BAD, PTEN, AKT, S6RP, and mTOR in adipocytes from IS and IR subjects.

## Discussion

Adipogenesis, the process of adipocyte differentiation and formation, plays a pivotal role in shaping metabolic health and insulin sensitivity (Morley et al., 2015). Understanding the molecular mechanisms underlying adipocyte differentiation is crucial for elucidating the etiology of adipogenesis-related pathologies and exploring potential therapeutic approaches. Our previous study has revealed that impairment of adipogenesis plays an important role in developing obesity-associated insulin resistance (Almuraikhy et al., 2016). In this study, we have investigated the relationship between impaired adipogenesis and insulin resistance in non-obese people. We also aimed to identify the molecular factors, including inflammatory markers and insulin signaling pathways, that may contribute to impaired adipogenesis. Additionally, we examined the ability of preadipocytes to undergo adipogenesis when exposed to TNF-α or metformin and insulin. In this study, preadipocytes were isolated from two subcutaneous depots (thigh and buccal fat pad), and the efficiency of proliferation and differentiation was first compared between them. The analysis showed no significant differences in proliferation percentage or adipogenic capacity between thigh- and buccal-derived cells, so data from both depots were subsequently pooled to maximize statistical power for detecting insulin-sensitive versus insulin-resistant and treatment effects.

Our results showed that preadipocytes derived from both IS and IR individuals exhibited comparable rates of proliferation. Moreover, TNF-alpha, a pro-inflammatory cytokine, inhibited the proliferation of preadipocytes from both IS and IR individuals. This suggests that preadipocyte proliferation is a process that may occur independently of insulin sensitivity. This is in line with Kulenkampff and Wolfrum (2019) who reported that a high-fat diet may induce preadipocyte proliferation and hyperplasia in specific adipose tissue depots, independent of insulin sensitivity. This distinction is important for understanding adipose tissue dynamics and developing therapeutic strategies for obesity and related metabolic disorders.

Our emerging results showed that the preadipocytes derived from IR individuals exhibited a reduced subcutaneous adipogenic capacity (by 38%, p = 0.0034) compared with those from IS individuals. This indicates that impaired adipogenesis plays a similar important role in the development of insulin resistance in non-obese individuals as previously shown in obese individuals (Almuraikhy et al., 2016). In parallel, our correlation analysis (Supplementary Figure S2) demonstrates that fasting triglycerides is the only clinical variable showing a clear and significant inverse correlation with adipogenic capacity, with higher triglyceride levels associated with poorer differentiation potential. Together, these findings indicate that impaired adipogenesis plays an important role in the development of insulin resistance in non-obese individuals and that elevated triglycerides likely represent a systemic biomarker of this underlying adipose dysfunction, rather than an isolated lipid abnormality. Based on this, the association between impaired adipogenesis and insulin resistance appears to be independent of obesity status and is accompanied by a triglyceride-rich dyslipidemic phenotype even in the absence of overt obesity. However, our results showed no significant differences in inflammatory cytokines and phosphorylation levels of proteins involved in insulin signaling between IS and IR. This could be explained by the fact that impaired adipogenesis in non-obese leads to dysfunctional adipose cells that are insulin resistant, even in the absence of increased inflammation, and that insulin resistance in adipose tissue is not solely determined by impaired insulin signaling. Moreover, the signaling assessment in our study was restricted to a limited panel of nodes, measured at one time point and under non-stimulated conditions. Classical features of adipocyte insulin resistance, such as reduced insulin-stimulated AKT phosphorylation or impaired downstream effects on glucose transport and lipogenesis, may therefore be present but not detectable under the basal conditions examined.

We have further measured the adipogenic capacity of preadipocytes under treatment with TNF-α and metformin from IS and IR participants. TNF-α is a pro-inflammatory cytokine that plays a key role in insulin resistance and metabolic disorders (Sethi and Hotamisligil, 2021). Treatment with TNF-α has decreased the adipogenic capacity in both IR and IS individuals. Notably, the reduced adipogenic capacity in response to TNF-α was more pronounced in in differentiating preadipocytes derived from IR individuals when compared to IS counterparts. This can be explained by the existing impairment in insulin signaling and chronic inflammatory state in the adipose tissue of IR individuals. These factors make preadipocytes more susceptible to the anti-adipogenic and insulin resistance-inducing effects of TNF-α, further exacerbating metabolic dysregulation.

These results were validated by the levels of IL-6 and IL-8 between untreated and TNF-α treated cells. IL-6 and IL-8 are key mediators linking adipose inflammation to metabolic dysfunction, and both have been implicated in impairing adipogenesis and promoting insulin resistance (Almuraikhy et al., 2016; Stoica et al., 2022). Significant differences in IL-8 were detected in IS, but not in IR individuals. Furthermore, TNF-α treatment had a more pronounced effect on IS cells, reflecting the higher baseline levels of these cytokines in insulin resistance status. Our results were further corroborated by the significant difference (p = 0.034) in serine phosphorylation of insulin receptor substrate-1 (IRS-1) between IR and IS when the cells are treated with TNF-α. Our data showed that the serine phosphorylation level was approximately 3 times higher in IR when compared to IS after treatment. Indeed, TNF-α induces insulin resistance through mechanisms such as increased serine phosphorylation of IRS-1, which impairs insulin receptor tyrosine kinase activity and insulin signal transduction (Sykiotis and Papavassiliou, 2001). Taken together, the differential response to TNF-α between IR and IS individuals in terms of adipogenic capacity can be largely explained by the heightened sensitivity of IR individuals to the insulin resistance-inducing effects of TNF-α, mediated through increased IRS-1 serine phosphorylation and the existing inflammatory state particularly IL-6 and IL-8 within their adipose tissues. This underscores the clear interplay between insulin resistance and adipocyte dysfunction. The absence of a significant difference in phosphorylation of IRS-1 between IR and IS when the cells are treated with metformin could be attributed to metformin’s primary actions occurring downstream or independent of IRS-1, rather than at this proximal adaptor. In particular, metformin can activate AMPK, modulate mTORC1 and mitochondrial function, and enhance glucose uptake through alternative pathways and mechanisms, thereby improving adipogenic capacity and insulin responsiveness without necessarily altering the basal IRS-1 phospho-signal detected in our study (Pernicova and Korbonits, 2014; Kalender et al., 2010).

Our results showed that metformin and insulin treatment increased significantly the adipogenic capacity of preadipocytes only in insulin resistance. In IR individuals, metformin treatment showed a marked improvement in adipogenic capacity, bringing it closer to levels observed in IS individuals and essentially normalizing their adipogenic function. Metformin treatment does not significantly increase adipogenic capacity further in IS individuals, suggesting a “ceiling effect” where additional stimulation does not lead to further enhancement once an optimal level is reached. These findings align with metformin’s known mechanisms of action, primarily targeting insulin resistance, and highlight its potential as a targeted therapy for improving adipose tissue function in insulin-resistant states (Dutta et al., 2023). By influencing gene expression, glucose transport, and enzymatic activity within adipocytes (Le Pelletie et al., 2021), metformin not only improves their insulin sensitivity but also enhances their adipogenic capacity.

Despite the small sample size of this study, partially driven by cell death of the samples, and the source of SAT samples (BFP and thigh), this study showed significant differences between the IR and IS groups. Another strength of this study lies in the unique selection of a non-obese and non-diabetic cohort, as opposed to the majority of the literature research where the focus is mainly on obese and diabetic patients. Although all participants were non-obese, IR group exhibited significantly higher mean BMI and fasting triglycerides compared to the IS group. These baseline differences represent potential confounders that may contribute to the observed impairment in IR adipogenic capacity, alongside insulin resistance *per se*. Given our limited sample size, multivariable adjustment was not feasible, so our findings might reflect the combined effects of insulin resistance and associated modest adiposity/dyslipidemia rather than isolated insulin resistance. Moreover, AST levels were modestly lower in IR group compared to IS controls, contrary to expectations from larger cohorts where insulin resistance and higher BMI typically associate with equal or elevated transaminases. In this small, non-diabetic cohort with normal liver biochemistry, the difference likely stems from random variation or unmeasured confounders such as recent physical activity, muscle mass variations, or subclinical factors influencing AST independently of metabolic status. Without concurrent ALT elevations or other liver injury markers, this finding warrants cautious interpretation and lacks mechanistic explanation from current data.

It is important to acknowledge that insulin signaling was evaluated in differentiated adipocytes derived from stromal vascular fraction cells, reflecting the insulin-responsive compartment of adipose tissue rather than undifferentiated adipose stem/progenitor cells (ASPCs). The lack of direct assessment of acute insulin responsiveness in non-differentiated ASPCs and the absence of glucose uptake measurements represent important limitations, particularly given that liver and skeletal muscle are major contributors to whole-body insulin sensitivity. Future work will therefore focus on interrogating insulin signaling in freshly isolated ASPCs from insulin-sensitive and insulin-resistant individuals. Nevertheless, we maintain that our results are robust and meaningful. This confidence stems from our methodological approach of collecting cells from individuals with confirmed insulin resistance and insulin sensitivity, which provides a strong foundation for our findings and conclusions. Our future research will also prioritize incorporating glucose uptake measurements to enhance the depth and interpretative power of our investigations.

## Conclusion

Impaired adipogenesis at subcutaneous adipocyte tissue plays a role in insulin resistance among non-obese individuals. The differential response to TNF-α and metformin in IR individuals, compared to their IS counterparts, suggests that targeting adipose tissue dysfunction and enhancing adipogenesis could be a promising therapeutic strategy against insulin resistance and its associated metabolic consequences, highlighting the need for a deeper understanding of the molecular mechanisms linking adipocyte phenotype and insulin resistance.

## Data Availability

The original contributions presented in the study are included in the article/Supplementary Material, further inquiries can be directed to the corresponding author.
