# Supplementary material for: The role of impaired adipogenesis in insulin resistance among non-obese individuals
Source: Front Physiol. 2026 Jan 7;16:1739215. doi: 10.3389/fphys.2025.1739215 (PMC12819270; doi:10.3389/fphys.2025.1739215)
Supplement: Supplementary file 1 [file DataSheet1.docx]

**Supplementary table 1:** Summary data for insulin signaling. Numbers represent Median Fluorescence Intensity (MFI).

| **Treatment** | **Depot** | **Group** | **p-GSK3a/b** | **p-PTEN** | **p-BAD** | **p-mTOR** | **p-P70S6K** | **p-S6RP** | **p-AKT** | **p-IRS1** |
| --- | --- | --- | --- | --- | --- | --- | --- | --- | --- | --- |
| UT | Thigh | IR | 94 | 68.5 | 123 | 34 | 105.5 | 46 | 60.5 | 57 |
| TNF-α | Thigh | IR | 61.5 | 38 | 81 | 28 | 65 | 35 | 59 | 46 |
| Metformin | Thigh | IR | 74 | 41 | 61 | 29.5 | 67 | 22.5 | 57 | 31 |
| UT | Thigh | IS | 66 | 42 | 82 | 25 | 69 | 33 | 68 | 53 |
| TNF-α | Thigh | IS | 327 | 418 | 122.5 | 61 | 227 | 594.5 | 87.5 | 104 |
| Metformin | Thigh | IS | 326 | 350 | 133 | 61 | 187 | 569 | 62.5 | 72 |
| UT | Thigh | IS | 144 | 456 | 320.5 | 57 | 187 | 3835 | 106.5 | 166 |
| TNF-α | Thigh | IS | 540 | 458.5 | 186 | 66.5 | 102 | 3267 | 55 | 101 |
| Metformin | Thigh | IS | 278 | 336.5 | 120 | 59 | 256 | 476 | 101 | 166 |
| UT | Thigh | IS | 72.5 | 32 | 95 | 21.5 | 77 | 47 | 58 | 39 |
| TNF-α | Thigh | IS | 62 | 43 | 73 | 31.5 | 82 | 23 | 61 | 40.5 |
| Metformin | Thigh | IS | 64 | 83 | 49.5 | 26 | 61.5 | 36 | 100 | 88 |
| UT | BF | IS | 992 | 903 | 760 | 128.5 | 169 | 785 | 114 | 174 |
| TNF-α | BF | IS | 406 | 308 | 339 | 89 | 218 | 820 | 93.5 | 204 |
| Metformin | BF | IS | 407 | 308.5 | 347 | 87.5 | 193 | 795 | 89 | 172 |
| UT | BF | IS | 54 | 41 | 141 | 31 | 81 | 44 | 58 | 47.5 |
| TNF-α | BF | IS | 379 | 695 | 187 | 54.5 | 141.5 | 521 | 81 | 93 |
| Metformin | BF | IS | 164 | 43 | 130 | 23.5 | 125 | 55 | 62.5 | 50 |
| UT | BF | IS | 84.5 | 27 | 112 | 27.5 | 87 | 256 | 74 | 62 |
| TNF-α | BF | IS | 68 | 35 | 82.5 | 23.5 | 68 | 25.5 | 58 | 40 |
| Metformin | BF | IS | 71.5 | 36 | 114 | 28.5 | 74.5 | 40.5 | 53 | 42 |
| UT | Thigh | IR | 509 | 556 | 177 | 66.5 | 104 | 1131 | 94 | 117 |
| TNF-α | Thigh | IR | 582 | 766.5 | 169 | 78.5 | 144 | 1066 | 78 | 140 |
| Metformin | Thigh | IR | 633 | 732.5 | 178 | 81 | 172 | 1144.5 | 91 | 129 |
| UT | BF | IS | 340 | 301 | 373.5 | 70 | 101 | 564 | 79.5 | 103 |
| TNF-α | BF | IS | 67 | 46 | 115 | 31.5 | 82 | 53 | 63 | 58 |
| Metformin | BF | IS | 471 | 219 | 426 | 61 | 113.5 | 634.5 | 75.5 | 73 |
| UT | Thigh | IS | 50.5 | 37 | 87 | 27 | 67.5 | 34 | 58 | 41 |
| TNF-α | Thigh | IS | 66 | 39 | 86 | 32 | 66 | 30 | 50 | 41 |
| Metformin | Thigh | IS | 60.5 | 42 | 90 | 34.5 | 79 | 36.5 | 58 | 41.5 |
| UT | BF | IR | 299.5 | 148.5 | 340 | 54 | 118 | 430 | 76 | 16 |
| TNF-α | BF | IR | 329 | 550 | 251 | 47 | 147 | 461.5 | 54 | 98 |
| Metformin | BF | IR | 387 | 473 | 219 | 51 | 104.5 | 1366.5 | 70.5 | 98 |
| UT | Thigh | IR | 58.5 | 33 | 99 | 32 | 58 | 33 | 75 | 51 |
| TNF-α | Thigh | IR | 786 | 1313 | 250.5 | 78.5 | 200.5 | 1371.5 | 100 | 289 |
| Metformin | Thigh | IR | 274.5 | 339 | 246 | 69 | 144.5 | 723 | 88 | 217 |
| UT | Thigh | IR | 69 | 37.5 | 88 | 31.5 | 83 | 28.5 | 63 | 48 |
| TNF-α | Thigh | IR | 480 | 420.5 | 191 | 66.5 | 127 | 666.5 | 85 | 125 |
| Metformin | Thigh | IR | 309 | 444 | 257.5 | 55.5 | 183 | 797 | 114 | 130 |
| UT | Thigh | IR | 57.5 | 32 | 76.5 | 33 | 72.5 | 38 | 61 | 52 |
| 4-HNE | Thigh | IR | 51.5 | 41 | 79.5 | 26 | 71 | 33.5 | 70.5 | 41 |
| TNF-α | Thigh | IR | 54.5 | 45.5 | 69 | 31 | 83 | 33.5 | 60.5 | 32 |


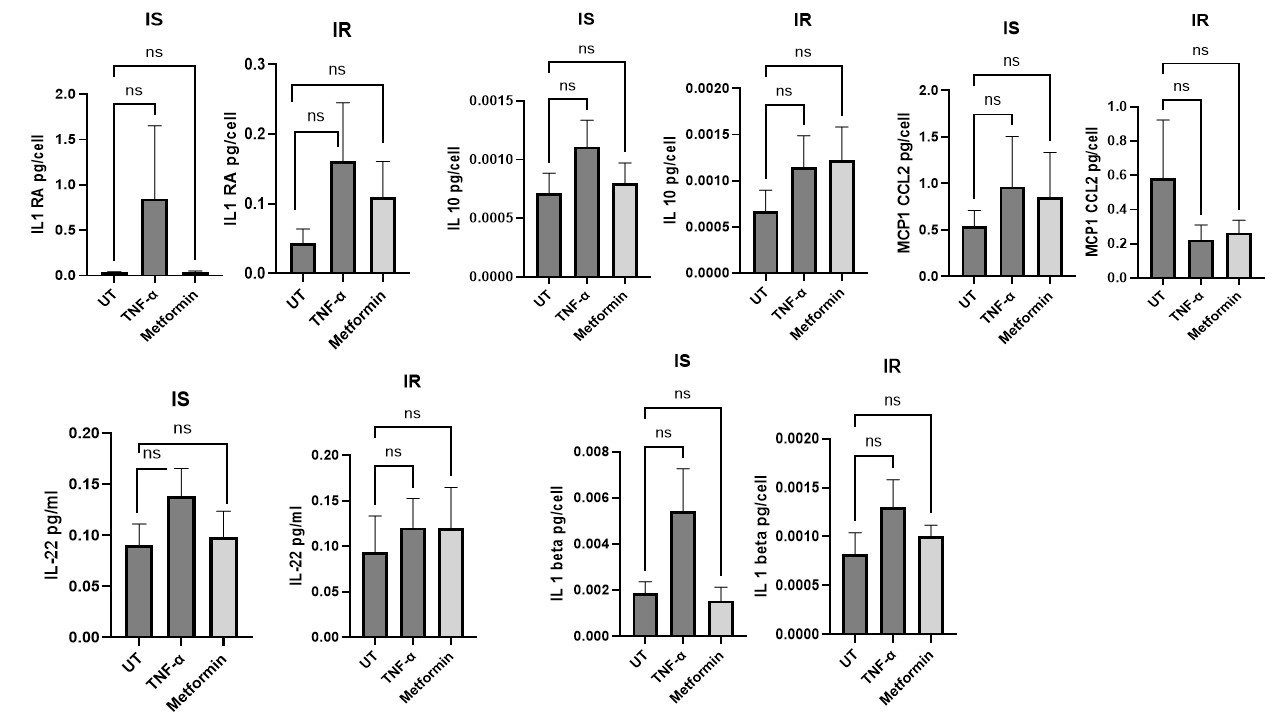


**Supplementary figure 1.** Level of secreted interleukins in IS and IR differentiated preadipocytes derived from lean/overweight individuals and after treatment with TNF-α or metformin. Cytokine levels were normalized by dividing cytokine levels to the cell number. Data are presented as mean ± SEM; Differences were tested by ANOVA/Kruskal Wallis followed by post-hoc Friedman/Dunnett test; *p < 0.05, ** p < 0.01.


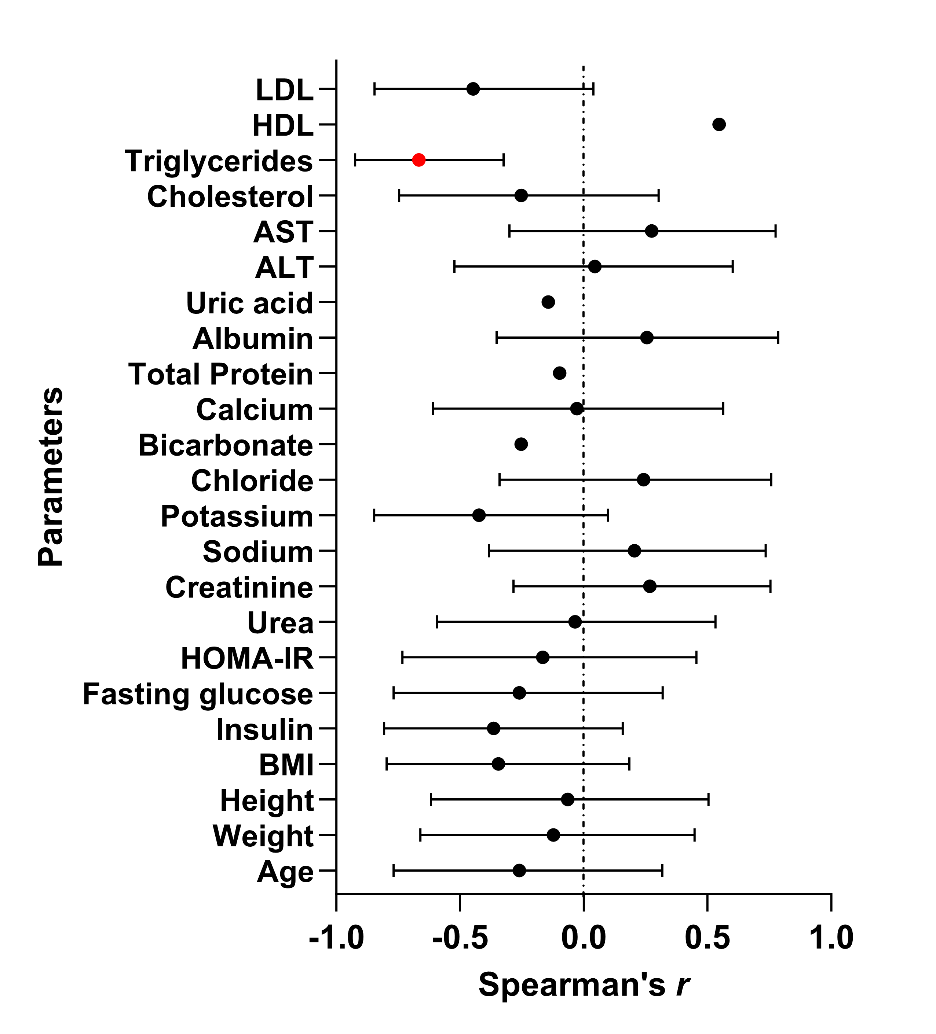


**Supplementary figure 2.** Spearman’s correlation between adipogenic capacity and clinical parameters. Red color denotes a p-value < 0.05 significance.
